# Comparison of pedicle screw fixation with or without cement augmentation for treating single-segment isthmic spondylolisthesis in the osteoporotic spine

**DOI:** 10.1038/s41598-023-27539-x

**Published:** 2023-01-16

**Authors:** Jian-cheng Peng, Hui-zhi Guo, Chen-guang Zhan, Hua-sheng Huang, Yan-huai Ma, Shun-cong Zhang, Yue-rong Xu, Guo-ye Mo, Yong-chao Tang

**Affiliations:** 1 Longgang Orthopedics Hospital of Shenzhen, Shenzhen, 518100 China; 2 The 1St Institute of Clinical Medicine, Guangzhou University of Chinese Medicine, 12 Airport Road, Baiyun District, Guangzhou, 510405 Guangdong China; 3 Spine Surgery Department, The First Affiliated Hospital of Guangzhou University of Chinese Medicine, Guangzhou, 510407 China

**Keywords:** Diseases, Medical research

## Abstract

The present study examined the necessity of cement-augmented pedicle screw fixation in osteoporotic patients with single-segment isthmic spondylolisthesis.Fifty-nine cases were reviewed retrospectively. Thirty-three cases were in the polymethylmethacrylate-augmented pedicle screw (PMMA-PS) group, and the other 26 cases were in the conventional pedicle screw (CPS) group. Evaluation data included operation time, intraoperative blood loss, hospitalization cost, hospitalization days, rates of fusion, screw loosening, bone cement leakage, visual analogue scale (VAS) scores, Oswestry disability index (ODI), lumbar lordosis (LL), pelvic tilt (PT) and sacral slope (SS).The operation time and blood loss in the CPS group decreased significantly compared to those in the PMMA-PS group. The average hospitalization cost of the PMMA-PS group was significantly higher than that of the CPS group. There was no significant difference in the average hospital stay between the 2 groups. The initial and last follow-up postoperative VAS and ODI scores improved significantly in the two groups. There were no significant differences in VAS and ODI between the 2 groups at each time point. The last postoperative spine-pelvic parameters were significantly improved compared with those preoperatively. In the PMMA-PS group, the fusion rate was 100%. The fusion rate was 96.15% in the CPS group. No significant difference was found between the two groups for the fusion rate. Nine patients in the PMMA-PS group had bone cement leakage. There was no screw loosening in the PMMA-PS group. There were 2 cases of screw loosening in the CPS group. There were no significant differences in screw loosening, postoperative adjacent segment fractures, postoperative infection or postoperative revision between the 2 groups. The use of PMMA-PS on a regular basis is not recommended in posterior lumbar interbody fusion for the treatment of single-segment isthmic spondylolisthesis with osteoporosis.

## Introduction

Population ageing is an important phenomenon for many countries worldwide. The prevalence of spinal degenerative diseases is increasing in ageing societies, and the number of patients undergoing spinal fusion surgery is increasing. Deyo et al.^1^ reported that the rate of lumbar spine fusion surgery in patients over 60 years in the United States increased 230% between 1988 and 2001. Rajaee et al.^2^ reported that the rate of spine fusion surgery in patients over 65 years in the United States increased by 239.2% between 1988 and 2008. Posterior lumbar pedicle screw fixation is an effective method to resolve lumbar degenerative disease, and it has the advantages of improving spinal stability and fusion rate^3^. However, osteoporosis often accompanies the natural ageing process of elderly individuals. Osteoporosis easily results in the loss of trabecular structure and an insecure screw-bone interface connection, which leads to the loosening and removal of screws and failure of internal fixation^4^. The loosening rate of conventional pedicle screws (CPSs) is approximately 60% in osteoporotic vertebral bodies^5,6^. How to enhance the stability of pedicle screws in the osteoporotic vertebral body and reduce the occurrence of internal fixation failures is a problem that orthopaedists must solve.

To improve the screw-holding force, a variety of methods have been reported to improve the screws, such as a cortical bone divergent trajectory, dual-threaded pedicle screws, expandable pedicle screws, larger diameter pedicle screws, extension pedicle screws, and polymethylmethacrylate-augmented pedicle screws (PMMA-PSs)^5–13^. PMMA-PSs are widely used in lumbar surgery. This technique has the advantages of improving the anti-extraction force of screws, and it achieves good functional outcomes and very low revision rates^14,15^.

Isthmic spondylolisthesis is a common spinal disease that requires a higher holding power of screws due to poor spinal stability and the need for pull-up and reduction during surgery^16,17^. Previous studies have reported that PMMA-PSs enhance the holding power of the screws^14^, but few cases have reported their use in single-segment isthmic spondylolisthesis in osteoporotic patients. When isthmic spondylolisthesis is combined with osteoporosis, the holding power of the screw is particularly important. However, does this procedure truly need to be used routinely in single-segment surgery? This research further studies this issue and reports the following findings.

## Materials and methods

Fifty-nine patients with single-segment isthmic spondylolisthesis combined with an osteoporotic spine who received posterior lumbar fusion and were followed up for a minimum of 2 years from January 2014 to December 2017 were reviewed retrospectively. Inclusion criteria: (1) Patients with single-segment isthmic spondylolisthesis who underwent posterior lumbar fusion; (2) Lumbar vertebral bone density measured using dual-energy X-ray absorptiometry, T value < − 2.5 SD; (3) Three months of conservative treatment that did not improve the symptoms, with indications for surgery; (4) Patients with I° or II° spondylolisthesis; and (5) Complete follow-up information. The exclusion criteria were as follows: (1) lumbar spine bone density test results that suggested normal bone mass or low bone mass; (2) patients who had undergone lumbar surgery and had III° lumbar spondylolisthesis; and (3) patients with severe cardiopulmonary and cerebrovascular disease. The same surgeons performed all the surgeries. Informed consent was obtained from each participant at the screening prior to any study-related activities being performed. The study was conducted at The First Affiliated Hospital of Guangzhou University of Chinese Medicine. It was reviewed by the appropriate ethics committee of The First Affiliated Hospital of Guangzhou University of Chinese Medicine and performed in accordance with the ethical standards laid down in an appropriate version of the 1964 Declaration of Helsinki.

Fifty-nine patients were divided into a PMMA-PS group and CPS group according to the presence or absence of bone cement around the screws. Thirty-three patients (7 males and 26 females; 64.67 ± 6.77 years old on average; average bone density − 3.35 ± 0.90 SD; 29.91 ± 9.15 m average follow-up time; surgical segment, 27 L4/5 cases and 6 L5/S1 cases) were in the PMMA-PS group, and the other 26 patients (8 males and 18 females; 60.27 ± 7.38 years old on average; average bone density − 3.25 ± 0.59 SD; 29.00 ± 8.32 m average follow-up time; surgical segment, 11 L4/5 cases and 15 L5/S1 cases) were in the CPS group. There was no significant difference in basic data between the two groups of patients (*P* > 0.05, Table 1).

**Table 1:** Basic data of two groups of patients.

	PMMA -PS group n = 33	CPS group n = 26	*P* value
Age (years)	64.67 ± 6.77	61.46 ± 6.26	0.067
Gender (male/female)	7/26	8/18	0.403
Bone mineral density (SD)	–3.35 ± 0.90	–3.25 ± 0.59	0.583
Follow-up time (m)	29.91 ± 9.15	29.00 ± 8.32	0.695
**Surgical segment**			
L4/5	27	11	1.000
L5/S1	6	15	

Spondylolisthesis is defined as slippage of the upper vertebral body relative to the lower vertebral body. All patients included here had I° or II° spondylolisthesis. I° indicates that the forwards sliding of the vertebral body does not exceed 1/4 of the sagittal diameter of the lower vertebral body. II° indicates the displacement of the vertebral body by more than 1/4 but less than 2/4.

### Operative methods and data collection

All patients received open posterior lumbar fusion surgery (TLIF). The use of annotation is decided by the preoperative BMD and the mechanical strength of the implanted pedicle screw. Bone cement is usually used when bone density is less than − 3.5. In the PMMA-PS group, the surgeon made an incision along the posterior midline approach during posterior lumbar fusion to reveal the pedicle access points in order and strengthened the cement nail channel under this perspective. The hollow pedicle screw was inserted first, and the bone cement was injected through the hollow screw. When the bone cement approached the posterior edge of the vertebral body, the surgeon stopped injecting bone cement. The surgeon injected approximately 0.1 ml of bone cement each time and injected approximately 1.5–2 ml of bone cement into a single nail channel. In the CPS group, TLIF was performed using a posterior midline open incision approach or a minimally invasive quadrant duct with a bilateral multifidus muscle approach. According to the need for decompression, the surgeon removed the corresponding lamina or facet joint and handled the intervertebral space. Intervertebral bone fusion was performed with autogenous bone or allogeneic bone, and an intervertebral fusion cage was placed in each segment. After the surgery, the patients performed routine lower limb functional exercises on the bed, and the drainage tube was removed when the drainage flow was < 50 ml/day. Patients wore a waist circumference to get out of bed after 3 or 4 days postoperatively and were required to wear a waist circumference for 1 month after surgery. Routine anti-osteoporosis treatment (oral calcium carbonate D3 tablet, 600 mg twice daily; zoledronic acid injection, 5 mg once annually via intravenous drip; patients with contraindications to zoledronic acid injection were changed to oral alendronate, 70 mg once weekly) was performed after surgery.

**Figure 1:**
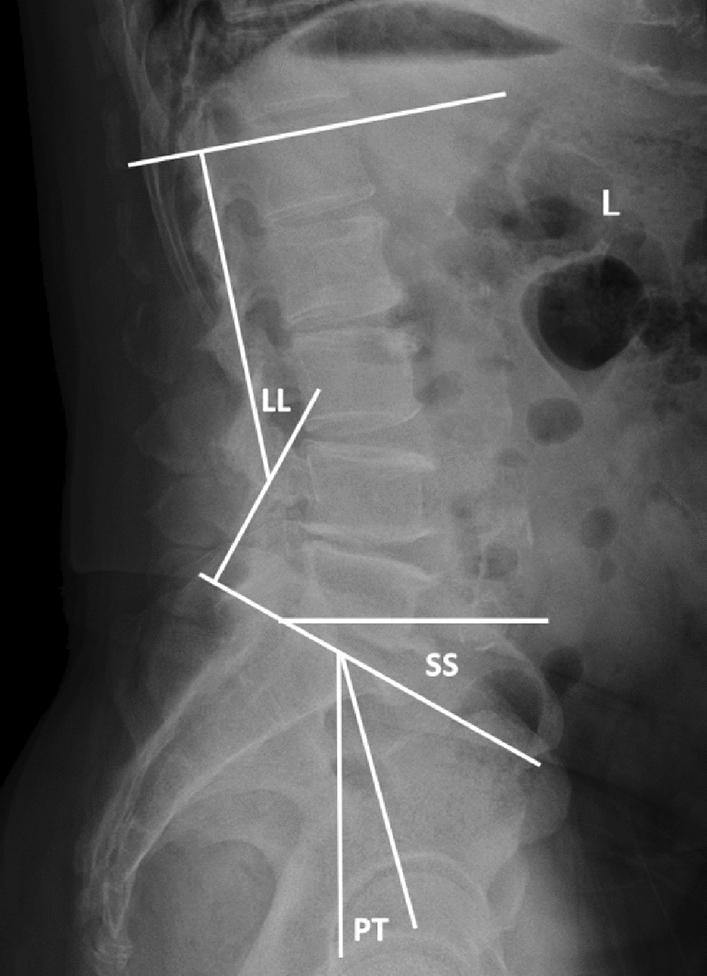
LL is defined as the angle between the end plate on L1 and the continuous end plate on S1.PT is defined as the angle between the connection and plumb line at the midpoint of the end plate and the center point of the femoral head on S1.SS is defined as the angle between the parallel and horizontal lines of the end plate on S1.

VAS and ODI scores of the two groups of patients were performed preoperatively, postoperatively and during the last follow-up to evaluate clinical efficacy. The operation time, intraoperative blood loss, surgical complications, hospitalization cost and hospital stay were recorded in the two groups. Lumbar spine X-ray and CT at the last follow-up were used to evaluate the existence of loose screws, bone cement leakage and failure of intervertebral fusion, LL, PT and SS (Fig. 1).

The VAS scoring procedure involved drawing a line on white paper, with an average scale of 10 segments marked as 0–10 points in turn, indicating a status ranging from no pain to severe pain. Patients independently chose one of the scores to indicate their current level of pain. The higher the score, the more severe was the pain.

The ODI scoring criteria were divided into 10 aspects, including pain intensity, quality of life, lifting, walking, and social life. Each problem was marked as 0–5 points from asymptomatic to serious, and the total score was 50 points. The higher the score, the more severe was the dysfunction.

The following criteria were used to determine whether intervertebral fusion was successful^18^. First, there was no relative displacement of the fusion segment on the X-ray film of lumbar hyperextension and flexion, or the intervertebral angle of the fusion segment was less than 3°. Second, there was no X-ray translucent band around the implant. Third, X-ray or CT showed visible bone tissue growth in or around the fusion cage and continuous cancellous bone bridges between the vertebral bodies of the fusion segment. If at least two of the above three indicators were met, intervertebral fusion was considered successful. The following criteria were used to determine the existence of loose screws^19^. First, the screws were displaced. Second, there was an X-ray translucent band greater than 1 mm around the screws. Third, CT showed undeveloped areas around the nail track. The presence of one or more of the above three indicators indicated loose screws.

### Statistical analysis

All data are expressed as the means ± standard deviations. The data were analysed using SPSS 23.0 software (IBM, Inc., Armonk, NY, USA). The operation time, intraoperative blood loss, and hospital stay of the two groups of patients were analysed using t tests of the summary sample. Preoperative, postoperative, and last follow-up postoperative LL, PT, SS, VAS and ODI were compared using t tests of paired samples. The chi-square test was used to compare fusion, screw loosening, postoperative adjacent segment fractures, postoperative infection and postoperative revision between the two groups of patients. P values < 0.05 were considered to be significant.

## Results

All patients underwent posterior lumbar fusion successfully. The amount of bone cement injected in the PMMA-PS group was 1.5–2 ml in a single nail channel. The operation time and intraoperative blood loss in the CPS group were significantly lower than those in the PMMA-PS group (*P* < 0.0.5, Table 2). The average hospitalization cost of the PMMA group was significantly higher than that of the CPS group (*P* < 0.0.5, Table 2). No significant difference in hospital stay was observed between the PMMA-PS and CPS groups *(P* > 0.0.5, Table 2). The postoperative and last follow-up postoperative VAS and ODI scores improved significantly in the two groups (*P* < 0.0.5, Table 3). There were no significant differences in VAS and ODI at each time node between the 2 groups (*P* > 0.05, Table 3). The last postoperative LL, PT and SS in the CPS group and PMMA-PS group were significantly improved compared with the preoperative period, and the sagittal balance was corrected (*P* < 0.0.5, Table 3).

**Table 2:** Surgery-related information for both groups of patients.

	PMMA-PS group (n = 33)	CPS group (n = 26)
Operation time (min)	211.58 ± 47.30	181.62 ± 41.33^①^
Intraoperative blood loss (ml)	425.76 ± 264.71	291.15 ± 137.06^②^
Hospitalization cost (yuan)	82,439.91 ± 3492.95	66,041.35 ± 1470.28^③^
Hospital stay (days)	16.91 ± 5.11	16.08 ± 5.93^④^

^①②③^Compared with PMMA-PS group, *P* < 0.05.

^④^Compared with PMMA-PS group, *P* > 0.05.

**Table 3:** LL, PT, SS, VAS and ODI of the two groups in the L4/5 and L5/S1 surgical segments.

	L4/5		L5/S1	
	CPS group	PMMA-PS group	CPS group	PMMA-PS group
**LL, degrees**				
Preoperation	43.41 ± 3.49	42.34 ± 4.39	44.08 ± 3.91	40.74 ± 3.40
Last follow up	47.96 ± 3.73	46.57 ± 5.04	50.28 ± 3.68	46.05 ± 4.02
*P* value	0.008	0.002	0.000	0.033
**PT, degrees**				
Preoperation	23.40 ± 2.32	23.29 ± 3.29	24.56 ± 1.89	22.21 ± 2.21
Last follow up	18.06 ± 2.27	18.13 ± 2.32	17.89 ± 1.90	17.17 ± 2.70
*P* value	0.000	0.000	0.000	0.005
**SS, degrees**				
Preoperation	32.16 ± 6.12	33.62 ± 6.37	30.28 ± 6.44	28.84 ± 3.30
Last follow up	38.55 ± 6.73	38.47 ± 7.08	36.23 ± 7.06	40.15 ± 1.92
*P* value	0.031	0.011	0.023	0.001
**VAS**				
Preoperation	6.09 ± 0.30	6.74 ± 1.10	7.27 ± 0.96	7 ± 0.89
Post-operation	1.55 ± 0.69^①^	1.63 ± 0.63^①^	1.80 ± 0.56^①^	2.17 ± 0.41^①^
Last follow up	0.66 ± 0.50^①^	0.67 ± 0.48^①^	0.80 ± 0.41^①^	1.17 ± 0.41^①^
**ODI**				
Preoperation	49.27 ± 7.11	50.53 ± 7.91	48.59 ± 5.57	51.33 ± 8.64
Postoperation	15.64 ± 5.28^①^	13.60 ± 3.22^①^	14.37 ± 4.26^①^	12.33 ± 2.66^①^
Last follow up	3.82 ± 0.98^①^	3.20 ± 1.52^①^	3.22 ± 1.60^①^	3.83 ± 1.60^①^

^①^Compared with preoperation, *P* < 0.05.

The fusion rate in the PMMA-PS group was 100%, and that in the CPS group was 96.15%, with no significant difference found between the two groups (*P* > 0.05, Table 4). Nine cases of bone cement leakage (27.27%) were found in the PMMA-PS group, including 3 cases of paravertebral vein leakage, 4 cases of anterior vertebral vein leakage, 1 case of anterior vertebral nail hole leakage, and 1 case of spinal canal leakage, which did not cause related complications. There was 1 case of postoperative adjacent segment fracture, 1 case of postoperative revision and no screw loosening or postoperative infection in the PMMA-PS group. There was 1 case of postoperative revision, 2 cases of screw loosening, 1 case of postoperative infection and no cases of postoperative adjacent segment fracture in the CPS group. There were no significant differences in screw loosening, postoperative adjacent segment fractures, postoperative infection or postoperative revision between the 2 groups (*P* > 0.05).

**Table 4:** The fusion status and postoperative complications of the two groups.

	PMMA -PS group (n = 33)	CPS group (n = 26)	χ^2^	*P* value
Fusion (n)	33/33	25/26	1.291	0.256
Screw loosening (n)	0/33	2/26	2.628	0.105
Fracture of adjacent segment	1/33	0/26	0.807	0.371

**Figure 2:**
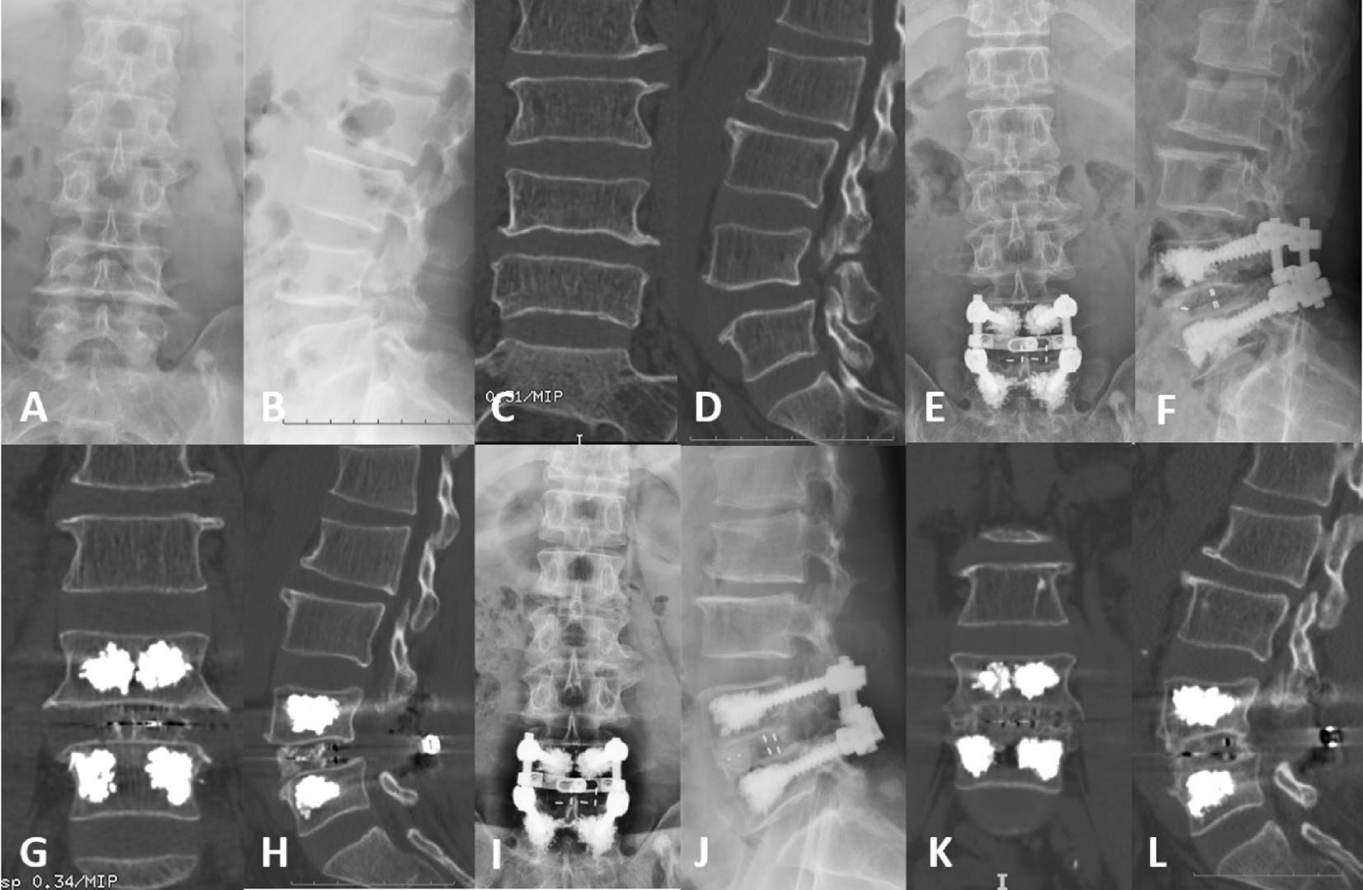
Radiological images of a representative case with PMMA-PS. (**A**–**L**) A 62-year-old osteoporotic male with isthmic spondylolisthesis in the L4/L5 segments. (**A**–**D**) Preoperative X-rays and CT showed forward slip of the L4 vertebra and L4 spondylolysis. (**E**–**H**) Postoperative X-ray and CT showed that the internal fixation position was good and that the bone cement was in the vertebrae. (**I**–**L**) Postoperative X-ray and CT at 36 months after fusion surgery showed that the internal fixation device did not loosen or break, the vertebra did not show slippage, the intervertebral fusion was good, the bone cement was well filled, and there was no obvious cement leakage.

**Figure 3:**
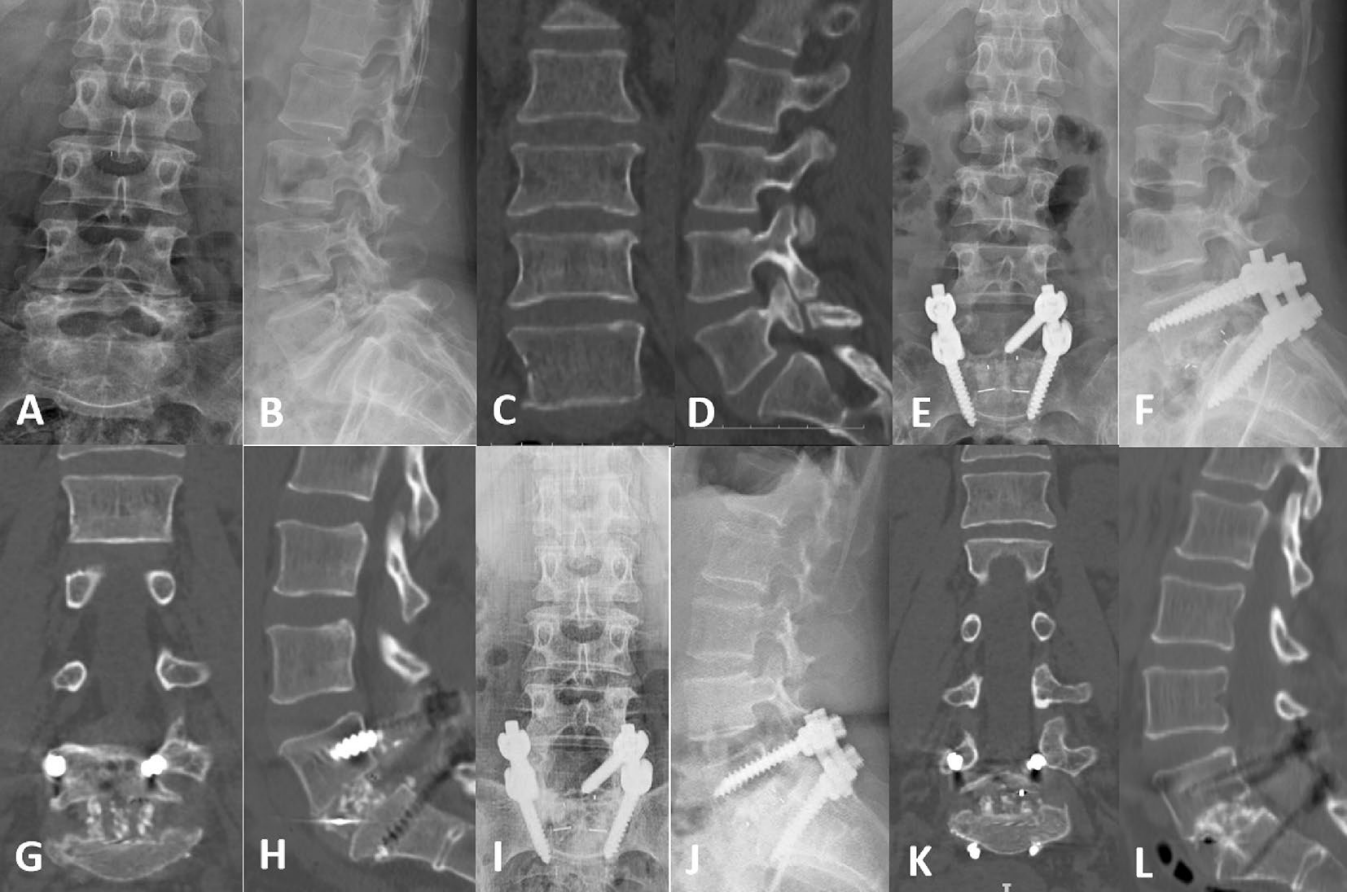
Radiological images of a representative case with CPS. (**A**–**L**) A 61-year-old osteoporotic female with isthmic spondylolisthesis in the L5/S1 segments. (**A**–**D**) Preoperative X-rays and CT showed forward slip of the L5 vertebra and L5 spondylolysis. (**E**–**H**) Postoperative X-rays and CT showed that the internal fixation position was good and that the L5 vertebra was well reset. (**I**–**L**) Postoperative X-ray and CT at 24 months after fusion surgery showed that the internal fixation device did not loosen or break, the vertebra did not show slippage, and the intervertebral fusion was good.

We present two representative cases: PMMA-PS (Fig. 2) and CPS (Fig. 3). The patients received good follow-up, including imaging and clinical evaluations.

## Discussion

CPSs are used as internal fixation instruments in spine surgery, and employing three-column fixation provides good segment stabilization and orthopaedic support. However, screw loosening easily occurs when osteoporotic patients undergo lumbar internal fixation due to the insufficient fixation strength of osteoporotic vertebrae. The literature states that the rate of pedicle screw loosening in osteoporotic vertebrae is approximately 60%, which is approximately 3–5 times higher than that observed in nonosteoporotic patients^5,6^. It is difficult for osteoporotic vertebrae to provide good pullout strength for pedicle screws due to the loss of bone mass and sparse bone trabeculae. Due to vertebral body forwards sliding and rotation, lumbar-pelvic sagittal imbalance and intervertebral instability^16,17^, the pullout strength of pedicle screws must be higher than that of pedicle screws during surgery, especially in isthmic spondylolisthesis. Biomechanical studies confirmed that the pullout strength of the pedicle screw was significantly reduced when the bone density was less than 0.777 ± 0.330 g/cm^2^^20^. Okuyama et al.^21^ reported that the maximum pullout strength of the pedicle screw decreased by 60 N for every 10 mg/cm^2^ decrease in bone density, and the pedicle screw did not have sufficient stability in the vertebral body when the bone density was less than 80–90 mg/cm^2^. Improving the pullout strength of pedicle screws in osteoporotic vertebral bodies is a problem that spine surgeons must face.

To prevent the failure of internal fixation of the lumbar spine due to osteoporosis, the following surgical options are primarily available. Some scholars have suggested that the pullout strength of cortical bone screws is 30–60% higher than that of traditional pedicle screws, which provides a feasible alternative method for traditional pedicle screws^22^. Wu et al.^5^ evaluated 157 patients undergoing lumbar fusion surgery, and the screw loosening rate in the expansion screw group (4.1%) was significantly lower than that in the ordinary screw group (12.9%). Biomechanical studies^11^ showed that the average pullout strength of double-threaded screws (2726.8 N) was significantly higher than that of hybrid-threaded screws (1890.2 N) and single-threaded screws (2213.3 N). However, some scholars^10^ proposed that double-threaded screws and ordinary pedicle screws showed the same axial extraction force and anti-fatigue strength. Studies have shown that larger diameter screws increase the pullout strength and that the pullout strength increases 35% when the screw diameter is increased by 2 mm^11,23^. PMMA-PSs are one of the most common ways to prevent the failure of internal fixation in osteoporotic lumbar internal fixation surgery. The use of bone cement significantly improves the pullout force and anti-fatigue resistance of screws. Biomechanical studies have reported that the pullout strength of PMMA-PS is 119%-213% higher than that of conventional pedicle screws^24,25^.

The application of PMMA-PSs has clear advantages in the surgical treatment of osteoporosis patients, but postoperative complications cannot be ignored. The most common complication is the leakage of bone cement, which may cause nerve damage, pulmonary embolism, anaphylactic shock and death^26–29^. Previous studies reported that the incidence of bone cement leakage in the strengthening of bone cement nail channels was 5.4–66.7%^18,30,31^. The present study found 9 cases (27.27%) of bone cement leakage in the PMMA-PS group, including 3 cases of paravertebral vein leakage, 4 cases of anterior vertebral vein leakage, 1 case of anterior vertebral nail hole leakage and 1 case of spinal canal leakage. There is no systematic study of the preventive measures of bone cement leakage in the strengthening of bone cement nail channels. Based on experience and the literature review of percutaneous vertebroplasty, the author summarizes the following measures to prevent bone cement leakage. First, the slow injection of 2–3 ml of dough-like bone cement into each anterior middle of the vertebral body under low pressure improves the pullout strength of the pedicle screw and prevents bone cement leakage^32,33^. Second, high-viscosity bone cement has a lower risk of leakage; however, the pressure of injection is higher, and the operating time is shorter^34^. Third, placement of the tip of the screw in the middle third of the vertebral body during screw insertion should be avoided to prevent bone cement from leaking into the spinal canal along the central vein of the vertebral body. If the bone cement approaches the posterior edge of the vertebral body or bone cement leaks, the bone cement injection should be stopped immediately. An average of 1.5–2 ml of bone cement was injected into each pedicle in the PMMA-PS group, and there was no symptomatic bone cement leakage.

When fusion internal fixation is performed for patients with osteoporotic lumbar spine disease, most of the literature recommends strengthening of the bone cement nail channel to prevent the risk of screw loosening and fracture after surgery. However, the research subjects^35–37^ were patients with multiple segments, mostly mixed with a single segment and double segment. Whether single segments must be strengthened is not clear. Nagahama et al.^38^ followed up 40 osteoporotic patients with lumbar spondylolisthesis who underwent single-segment PLIF and found that the fusion rate in the bisphosphonate group was as high as 95% 1 year after surgery, that in the vitamin D group was only 65%, and 24% of the patients had fracture of the adjacent vertebral body in the vitamin D group. Chen et al.^39^ followed 79 osteoporotic patients with lumbar spondylolisthesis who underwent single-segment fusion and found that the fusion rates of the zoledronic acid group and the nonzoledronic acid group were greater than 82% on the basis of taking calcium and vitamin D regularly, but the latter group had a high incidence (17%) of fracture in adjacent segments. Fischer et al.^40^ performed a literature review and reported that teriparatide increased bone mass and promoted intervertebral fusion. Therefore, single-segment fusion internal fixation also achieved a higher fusion rate for cases of lumbar spondylolisthesis combined with osteoporosis with the cooperation of regular anti-osteoporosis treatment, and no significant internal fixation failure was observed. The results of the present study showed that the PMMA-PS group (100%) and the CPS group (96.15%) had satisfactory fusion rates, and there was no significant difference between the two groups. There was 1 case of postoperative adjacent segment fractures, 1 case of postoperative revision and no screw loosening or postoperative infection in the PMMA-PS group. There was 1 case of postoperative revision, 2 cases of screw loosening, 1 case of postoperative infection and no postoperative adjacent segment fracture in the CPS group. There were no significant differences in postoperative fusion, screw loosening or postoperative adjacent segment fractures between the 2 groups. The situation may be related to the combined use of zoledronic acid on the basis of regular anti-osteoporosis treatment in postoperative patients.

In lumbar internal fixation surgery, postoperative intervertebral fusion is highly important. Intervertebral fusion is prone to screw loosening and breaking. especially in patients with osteoporosis^4–6^. Therefore, when osteoporotic patients with lumbar spondylolisthesis undergo lumbar internal fixation and fusion surgery, it is necessary to pay attention to the relevant factors of intervertebral fusion. Okuda et al.^41^ retrospectively analysed 101 patients with lumbar spondylolisthesis through at least 3 years of follow-up and found that the incidence of delayed fusion was significantly higher in patients over 70 years of age than in patients under 70 years of age, but age did not affect the clinical efficacy. Park et al.^42^ studied 881 intervertebral spaces in 784 patients who were treated with TLIF and found that the pear-shaped intervertebral space easily caused backwards movement of the cage and affected intervertebral fusion. Abbushi et al.^43^ analysed 40 patients with lumbar spondylolisthesis who underwent lumbar fusion surgery and found that bullet-shaped cages, cages in the central vertebral body, an insufficient cage height, stress in the posterior column, and endplate damage were risk factors that led to postoperative fusion failure. Kimura et al.^44^ followed up 1070 patients who underwent PLIF, including 76 patients with isthmic spondylolisthesis, and suggested that because of the angle and pear shape of the intervertebral space, L5/S1 had a large degree of spatial mobility, which easily caused the cage to move backwards. To counteract the effects of the abovementioned factors on intervertebral fusion, based on our experience and literature review, we performed the following measures to avoid risks. First, cages were not placed in the middle of the weak endplate, especially in patients with osteoporosis and pear-shaped intervertebral spaces. Second, damage to the cartilage endplate was avoided during the surgery. Third, the size of the cage used was slightly larger than the size measured during the surgery. When the fusion segment was L5/S1, an angled cage was selected, which fit the upper and lower endplates such that the cage obtained a larger weight-supporting area, which reduced the loosening rate of the screws and promoted fusion^45^.

The data of the present study showed that the operation time and intraoperative blood loss in the CPS group were lower than those in the PMMA-PS group, but there was no significant difference in the length of hospital stay between the two groups. The higher blood loss in the PMMA group may have been related to the longer operation time. The last postoperative LL, PT, and SS of all patients were significantly improved compared with those before surgery, and the sagittal balance was corrected. The clinical symptoms of the two groups of patients improved significantly compared to those before surgery, which was related to nerve decompression, reduction of spondylolisthesis and improvement of spine-pelvic parameters. At the final follow-up, there were no significant differences in VAS or ODI scores between the two groups.

There are several deficiencies in this study. First, it was a single-centre retrospective study, and more prospective investigations are needed. Second, the follow-up time was short, and the sample size was small. Only patients with I° and II° lumbar spondylolisthesis were included, and cases with III° lumbar spondylolisthesis were not included, which may have biased the conclusions. Third, the sequence of bone cement injection and screw placement was not clear.

## Conclusions

When osteoporotic patients with single-segment isthmic spondylolisthesis undergo lumbar fusion internal fixation, the use of PMMA-PSs achieved similar clinical effects as CPSs. Ordinary pedicle screws have the advantages of avoiding postoperative bone cement leakage, less operation time, and less intraoperative blood loss. Routine use of PMMA-PSs is not recommended in osteoporotic patients with single-segment isthmic spondylolisthesis. Patients with osteoporosis must pay attention to regular anti-osteoporosis treatment after lumbar fusion internal fixation. Treatment with bisphosphonates and teriparatide is necessary. When using PMMA-PSs, the amount of bone cement injected should be strictly controlled. When bone cement approaches the posterior edge of the vertebral body or bone cement leaks, the bone cement injection should be stopped immediately.

## Data Availability

The datasets used and/or analysed during the current study available from the corresponding author on reason able request.
